# Research on the Influence of Network Position on Corporate Social Responsibility: Moderating Effect Based on Ownership Concentration

**DOI:** 10.3389/fpsyg.2022.894725

**Published:** 2022-05-16

**Authors:** Liang Qu, Yuanjie Xu, Yajing Guo

**Affiliations:** School of Business Administration, Zhejiang Gongshang University, Hangzhou, China

**Keywords:** interlocking directorate network, centrality, structural hole, corporate social responsibility (CSR), ownership concentration

## Abstract

Based on the social network theory and the institutional theory, this study examines the influence of corporate network position on corporate social responsibility (CSR), and further explores the moderating role of ownership concentration. Given the characteristics of CSR in different aspects, this study explores the relationship between corporate network position and economic CSR, environmental CSR, and social CSR from the two aspects of the centrality and structural holes of interlocking directorate network based on the data of 1,034 Chinese A-share listed companies from 2010 to 2019. The results show that the centrality and structural holes of interlocking directorate network have positive effects on the overall level of CSR, and the impacts on economic CSR and environmental CSR are stronger than that on social CSR. In addition, ownership concentration has a positive moderating effect on the relationship between corporate network position and CSR. These findings enrich the depth of research on CSR, clarify the influence of the characteristics of interlocking directorate network on CSR in different dimensions, and supplement the knowledge of existing research.

## Introduction

All enterprises are in the social network, and the interlocking directorates are the bridge connecting an enterprise with others. Directors who hold directorships in both companies at the same time are called interlocking directorates (Mizruchi, 1996). Interlocking directorates play an important role in information exchange and the diffusion of business practices. As interlocking directorates hold directorships in more than one enterprise, they form a social network among the companies they serve, that is, interlocking directorate network, which constitutes a reliable and low-cost information transmission mechanism among enterprises (Haunschild, 1993). Meanwhile, the nature of a small world network of interlocking directorates network shows that the speed of information diffusion among enterprises is fast and the efficiency of obtaining resources is high in the formed interlocking directorate network (Newman and Strogatz, 2001; Battiston, 2004; Conyon, 2006; Durbach, 2009; Prem Sankar and Asokan, 2015; Sankowska, 2016). In addition, many studies (Chiu and Teoh, 2013; Srinivasan and Wuyts, 2018) have proved that interlocking directorates have played an important role in the profitability (Larcker and So, 2013; (Ortiz-de-Mandojana and Aragon-Correa, 2015), innovation ability (Zaheer, 2005; Huang and Zhang, 2020), and the ability to cope with changes in the external environment of enterprises (Carpenter, 2001; Ortiz-de-Mandojana et al., 2012; Martin and Gözübüyük, 2015). For the interlocking directorate network, this study examines the impact of the position of interlocking directorate network on corporate behavior from the two dimensions of centrality and structural hole according to the practices of Martin and Gözübüyük (2015) and Wang et al. (2019b).

The emergence of interlocking directorates has made relationship network become an important way for enterprise development and an important influencing factor to fulfill their corporate social responsibility (CSR) (Besser, 2011). Sheldon (1924) proposed CSR first and believed that the concept of “shareholder first” which has always been supported by managers is no longer suitable for the current goal of enterprise development. In addition to paying attention to the interests of shareholders, enterprises should pay attention to the interests of employees, government, community, environment and other groups as well. CSR is that enterprises coordinate their own interests with social interests to realize the common sustainable development of enterprises and society. Normally, CSR behavior includes charitable donations, social assistance, environmental protection, etc. (Roeck et al., 2014), and we emphasize the overall contribution of enterprises to stakeholders, environment and society in production and operation. From the meaning of CSR in this study, it can be seen that CSR emphasizes meeting the expectations of multiple stakeholders (Aguinis, 2012). Therefore, we make an in-depth study from three dimensions, namely, economic CSR, environmental CSR, and social CSR.

In recent years, the results of studies on CSR have similarly shown that interlocking directorate network also affects the fulfillment of CSR (Ortiz-de-Mandojana and Aragon-Correa, 2015). In terms of the relationship between interlocking directorate network and CSR, most studies emphasize that interlocking directorates are conducive to improving the level of CSR (Ortiz-de-Mandojana et al., 2012; Mandojana and Aragon, 2015). However, some studies hold different opinions (Ben Barka, 2015). Marquis (2013) found that the characteristics of management and directors will affect the charitable donation of enterprises through the analysis of top 500 enterprises, and the board structure restricts the charitable behavior of company members. The divergence of existing research conclusions led scholars to investigate the possible impact of organizational boundary conditions. Martin and Gözübüyük (2015) took industry uncertainty as a moderating variable to explore the relationship between interlocking directorate network and firm performance. The research of Zona and Gomez-Mejia (2018) indicated that interlocking directorates may exert either a positive or a negative effect on firm performance, depending on the firm's relative resources, power imbalance, ownership concentration, and CEO ownership. Therefore, on the basis of exploring the impact of centrality and structural hole of interlocking directorate network on the three dimensions of CSR, we add the moderating effect of ownership concentration, to investigate the role of the internal relationship structure of the board of directors on the relationship between interlocking directorate network position and corporate behavior.

Specifically, we use the multiple regression method to explore the influence of interlocking directorate network on CSR behavior from the perspective of dual attributes of social network—centrality and structural hole. This method can intuitively explore the correlation between the two. To a certain extent, interlocking directorate network determines the future of an enterprise (Chuluun and Prevost, 2017), and more and more enterprises also rely on their corporate image in the “circle of friends” to strengthen their comprehensive strength and improve their competitiveness (Dass et al., 2014). Supported by Freeman's (1978) social network theory and Burt's (1992) structural hole theory, enterprises with high centrality have more ties with other enterprises in the network, which are easier to obtain key information; and the enterprises with more structural holes are in the key “hub” position in the network and have the right to dominate the information obtained. Therefore, to further explore the impact of interlocking directorate network on CSR, the first purpose of this study is to examine the impact of interlocking directorate network on CSR from two aspects: network centrality and network structural hole.

Next, we discuss whether there are differences on the influence of interlocking directorate network on enterprise economic CSR, environmental CSR, and social CSR. Corporate social responsibility is essentially a multi-dimensional concept (Carroll, 1991), and enterprises should also meet environmental and social requirements (Elkington, 1998) when pursuing economic benefits. Economic CSR involves the interests of the enterprise's direct stakeholders and is closely related to the enterprise's economic development (Carter, 2002). Environmental CSR promotes the enterprises to be more environment-friendly, which not only affects the reputation of enterprises but also helps enterprises to gain the legal recognition of other enterprises, to promote good cooperation among enterprises (Buysse, 2003), to gain the recognition of consumers and other stakeholders, and to enhance the brand recognition of enterprises and increase consumers' purchase intention. Social CSR is the expectation of the government, the public and the media, which covers social charitable donations, community activities, etc. It does not increase the interests of the enterprise directly, and this is different from the effect of economic CSR and environmental CSR. Therefore, it is necessary to explore the relationship between interlocking directorate network and different dimensions of CSR.

In addition, we also discuss the moderating role of ownership concentration in the relationship between interlocking directorate network and CSR. Shropshire (2010) believes that the relative power of the board of directors and the CEO will affect the role of interlocking directorates in the enterprise, which is especially reflected in that the duality of CEO and chairman has a significant impact on firm performance (Duru and Iyengar, 2016). In some enterprises, the board system is nothing but an empty shell, which is difficult to play important roles. As the directors failed to give full play to their functions of providing important suggestions and consulting for enterprise strategy, the possibility of interlocking directorates playing an active role decreased. Therefore, the setting of corporate leadership structure has become an important factor affecting the role of interlocking directorates in CSR. Although China has strengthened the supervision of major shareholders, the phenomenon of high concentration of ownership structure is still common. As for the influence of major shareholders on enterprises, the existing views mainly focus on the “incentive effect” and the “entrenchment effect” (Claessens and Djankov, 2000). In the process of interlocking directorates influencing CSR, what role does the major shareholders play, and whether the relationship between interlocking directorates and CSR is based on the incentive effect or the entrenchment effect needs to be further explored. Ownership concentration is the most common measurement of ownership structure, and the higher the ownership concentration, the higher the shareholding ratio of major shareholders. This determines whether the shareholders have the right to make decisions and whether they have the right to dominate the resources. Therefore, it is necessary to directly discuss the moderating effect of ownership concentration, so as to further our understanding of the differences in the impact mechanism of CSR of corporates with different ownership structures in the interlocking directorate network. Also, we further test whether the ownership concentration of corporates determines the relationship between interlocking directorate network and CSR.

This study makes several contributions to related research. First, we explore the impact on CSR from the two perspectives of the centrality and structural hole of the interlocking directorate network, which theoretically enhances the explanation of the internal mechanism of the research variables. Few scholars directly explore the relationship between interlocking directorate network and CSR. This study enriches the relevant research. Second, we also test the moderating effect of ownership concentration. Corporates with high ownership concentration have stronger motivation to undertake CSR behaviors, which deepens our understanding of the boundary conditions of the influence of interlocking directorate network on CSR. Third, the content of CSR in the existing literature (Sun et al., 2020) is relatively incomplete and does not take into account the internal differences of CSR. This study subdivides CSR into three dimensions, namely, economic CSR, environmental CSR and social CSR, which is conducive to distinguish the differences between different dimensions of CSR and overcome the possible errors caused by the overall concept, so as to explore the impact of interlocking directorate network on different dimensions of CSR. Also, this provides a guidance and suggestions to enterprises participating in CSR, and also further enriches the literature on CSR.

The rest of this study is detailed as follows: We put forward the research hypothesis on the basis of theoretical analysis at first. Then the research methods and empirical results are described in detail. The “Discussion” section elaborates on the theoretical and practical significance of this article, and eventually the limitations of the study and new directions for the future research are detailed.

## Theoretical Background And Hypotheses

Wellman (1988) proposed social network and believed that network is a series of social relations connecting participants, who have formed a relatively stable social structure. Social networks are closely related to access to business knowledge, information, and other resources. The location of network members is different, and the ability to obtain a variety of rare resources is also various. The way and efficiency of resource flow would be affected by the quantity, density, and intensity of social relations and the position of individuals in the network. As an invisible bridge between enterprises, interlocking directorates are important human capital and social resources, who have high professional quality and professional skills. Mizruchi (1996) proposed the interlocking directorate network in his research, and then it was quickly recognized by most scholars. It is generally believed that an interlocking directorate network among enterprises is one of the main channels of information transmission and exchange in the process of rapid social development (Chiu and Teoh, 2013). The interlocking directorate network formed by the relationship of part-time directors is full of rich social capital and information resources, which has an important impact on the operation and management decision-making of enterprises.

As for the position of enterprises in the interlocking directorate network, most of the existing studies describe it from the following two aspects: Centrality and structural hole. “Centrality” means whether the enterprise is in the center or edge of the network, and “structural hole” refers to the discontinuity between some nodes in the network (Martin and Gözübüyük, 2015). On the one hand, centrality measures the importance of individuals in the network, and concretizes the degree of enterprises acting as the central hub of the network and the degree of resource acquisition and control (Haunschild, 1998). On the other hand, the focus of structural hole is different from that of centrality. Structural hole does not emphasize direct connection, but pays more attention to the relationship mode with self-connected enterprises. That is, if an enterprise can connect the enterprises that cannot be directly connected, it indicates that this enterprise occupies the position of the structural hole in the interlocking directorate network (Burt, 1992). This study explores the impact on CSR from the following two aspects: Centrality and structural hole.

### Interlocking Directorate Network and CSR

Social network theory holds that the strategic decision-making of enterprises is affected by the social network embedded in enterprises (Granovetter, 1985), which is embodied in the information acquisition, social behavior, innovation output, and so on. The position in the network reflects the control and influence of the enterprise, and has an impact on the efficiency of obtaining information and resources, thus affecting the behavior of the enterprise. The centrality of interlocking directorate network is a variable to measure whether individuals are easy to be noticed in the network and whether their position is critical. The mutual imitation and learning among corporates in the network is the result of information transmission and resource sharing, which leads to the “peer effects” of corporate behaviors (Hallock, 1997; Kang, 2008; Bizjak and Lemmon, 2009; Yang, 2011; Chiu and Teoh, 2013). A study on stock market migration reveals that strong ties to in-group members reduced the impact of identity-discrepant cues, while strong ties to out-group members enhanced the impact (Rao and Davis, 2000). Meanwhile, the enterprise in the central position reflects a strong ability to capture key information in interlocking directorate network. The higher the network centrality of the enterprise, the higher the exposure of the enterprise in this “circle.” Normally, enterprises will choose other enterprises with good reputation to cooperate instead of those with bad reputation, so as to protect their reputation from the influence of enterprises with bad reputation. As pressure from relevant enterprises on CSR, the target enterprises actively fulfill CSR to obtain the legal recognition of relevant enterprises and maintain and strengthen the relationship with important stakeholders (Buysse, 2003). Whether to increase the interests of shareholders, donate to the society or participate in charity activities, the role of CSR is to gain the recognition of the government, media, and stakeholders (Robinson and Irmak, 2012; Jones and Willness, 2014), so as to obtain a positive evaluation of the corporate image and reputation.

Institutional theory holds that organizations in the institutional environment will inevitably be under the pressure of the institutional environment (Meyer, 1977). To obtain the legitimacy recognition of its stakeholders, enterprises must abide by the institutional pressure brought by the institutional environment and take actions that can obtain legitimacy (Meyer, 1977). In other words, social network has a restrictive effect on the behavior of the participants in the network, and enterprises in the center of the network will be subject to more pressure from all aspects (Wang, 2011). This urges enterprises to actively undertake CSR to maintain a good corporate image (Li et al., 2015a). When enterprises appear in the public view with a very high frequency without timely CSR behavior, the public and the media will make bad comments on their behavior, resulting in bad reputation and image. Other enterprises cooperate with them will leave as well, gradually damaging the company's business performance. Therefore, we hold that enterprises with higher network centrality will actively fulfill their CSR due to the influence of “reputation mechanism.”

**Hypothesis 1a:** There is a positive relationship between the centrality of interlocking directorate network and the level of CSR.

The importance of an enterprise in the network depends not only on the number of enterprises it is directly connected to but also on whether it is in a key position in the network that controls the transmission of information, that is the number of structural holes owned by the enterprise. The directors in key transmission positions have the right to choose when to start and to end the exchange of information among enterprises, as well as the content of the exchanged information (Burt, 2000; Markóczy et al., 2013). Enterprises with structural holes can connect the unconnected enterprises in the network so as to shorten the information transmission path between enterprises, speed up the flow of information, and promote the dissemination and utilization of resources (Uzzi, 1997). Compared with the enterprises at the edge of the interlocking directorate network, the enterprises occupying the position of structural holes have more competitive advantages, which can obtain information advantage and control advantage by manipulating the structural holes, so they occupy the dominant position (Burt, 1992). The advantage of information is that enterprises occupying the position of structural holes can significantly improve the efficiency of information transmission in the case of uncertain business environment, which is conducive to the learning of advanced technology and management experience (Mol, 2001); the advantage of control comes from the fact that enterprises occupying the position of structural holes can effectively control the information flow between different enterprises and selectively arrange the information of surrounding enterprises, that is, control the content, time, and quantity of information sharing (Gilsing et al., 2008). In addition, the richness of structural holes emphasizes the number of “non-redundant” connections of enterprises (Burt, 1992). The resources and information obtained by the enterprises occupying the position of the network structural holes and those in the network center are heterogeneous, and the enterprises in the position of the structural holes can obtain more non-redundant information. That is, the higher the level of the structural gap for a company in the interlocking directorate network, the more redundant information inflow is reduced. When the enterprises are in the position of structural holes, they can grasp the initiative of resource flow and have the power to control the information exchange and resource transmission between the individuals directly connected to them, while the peripheral enterprises do not have the abilities. Therefore, enterprises with more structural holes have higher control and intermediary abilities, and the necessity and motivation to undertake CSR will be greater and stronger.

**Hypothesis 1b:** There is a positive relationship between the structural hole of interlocking directorate network and the level of CSR.

### Interlocking Directorate Network and CSR in Different Dimensions

There are differences in the impact of different dimensions of CSR on the interests of affiliated companies. Economic CSR involves the interests of the corporate's shareholders, employees, customers, suppliers, and other direct stakeholders, which is the expectation of these stakeholders (Carter, 2002; Buysse, 2003). Studies (Larcker and So, 2013; Kaustia, 2015) have shown that enterprises in interlocking directorate network will obtain information through their own network advantages, reduce the environmental uncertainty faced by enterprises, promote cooperation between enterprises, and finally achieve the purpose of improving enterprise economic benefits. Enterprises in the center of the network often have many direct connections with other enterprises, so they can fully and timely obtain key information and have absolute influence to make its affiliated enterprises to imitate (Leary, 2014). At the same time, enterprises in the structural hole position will also gain the trust of edge enterprises by information and control advantages, thereby improving their business performance. In short, the fundamental purpose of performing economic CSR for enterprises is to improve the economic benefits of the enterprise, and the performance of economic CSR is the most direct short-term behavior related to the economic benefits of enterprises.

Social CSR covers donation, charity and other activities to meet the expectations of indirect stakeholders such as the community, the public, and the government. The research shows that the impact of donation on enterprises shows that the cumulative excess rate of return of the enterprise has increased significantly (Wang, 2011). It can be seen that enterprises can send positive signals to indirect stakeholders by fulfilling social CSR, so as to obtain goodwill and trust. When the peripheral companies cannot directly connect with the companies in the center of the network or in the structural hole position, they often judge whether the enterprise is a trustworthy organization and whether they can establish long-term cooperative relations with it later through the fulfillment degree of social CSR. Enterprises in the center of the network are closely connected with the surrounding enterprises and have higher influence and visibility. In addition to having a strong influence on other enterprises, enterprises with “high visibility” often get more attention from others (Zhang and Marquis, 2016). The government and the public hope that such enterprises can play an exemplary role, so they will have more and higher expectations that enterprises will take on more social CSR for the masses and the country (Wang et al., 2019b). Therefore, to get the support of other enterprises and the government, such enterprises will be more willing to fulfill social CSR.

Environmental CSR mainly refers to improving technology, reducing pollution, and making enterprises develop toward environment-friendly focus. The legitimacy theory holds that enterprises will be expected by responsibility from upstream and downstream enterprises. If downstream enterprises do not have enough awareness and corresponding actions on environmental protection, it is difficult to win the favor of suppliers, and actively fulfilling environmental CSR will continuously improve the willingness of suppliers to cooperate. At the same time, from the perspective of suppliers, suppliers usually do not choose to cooperate with enterprises with environmental reputation stains or scandals to protect their reputation. Therefore, suppliers, upstream and downstream enterprises, and investors will actually put pressure on the environmental CSR of the target enterprise. The attention and brilliance of public opinion brought by the influence of enterprises in the central position of the network or occupying the position of structural holes also make enterprises have to fulfill their environmental responsibilities. Furthermore, social norms and institutional theory regards legitimacy as the requirement for organizations to follow reasonable norms (Besser, 2011), which urges enterprises to actively fulfill environmental CSR to obtain the trust of peripheral companies and related resources. In addition, it is a long-term behavior for enterprises to undertake environmental CSR. The public may pay increasing attention to environment with the country's admiration for the concept of national green development. Moreover, the laws and regulations also urge enterprises to fulfill their environmental CSR to a greater extent. Compared with the CSR of other dimensions, environmental CSR is closely related to national policies. China aims to achieve carbon peak and carbon neutralization, so environmental CSR is more prominent with media and government supervision. Therefore, the closer to the network center, the stronger the supervision of environmental CSR, and the stronger the social pressure to fulfill environmental CSR. The enterprises in the center of the network and occupying the position of structural holes usually respond positively and implement the corresponding environmental CSR behavior.

**Hypothesis 2:** The centrality and the structural hole of interlocking directorate network have different impact intensity on different dimensions of CSR, and on what dimension of the CSR does the position in the interlocking directorate network have the strongest impact?

### Interlocking Directorate Network, Ownership Concentration and CSR

The agency theory holds that major shareholders have more motivation and ability than minor shareholders to supervise the management and operation activities of the corporate to promote the growth of the corporate's value under the same conditions (Shleifer, 1986). Based on the consideration of the long-term interests of the enterprise, the major shareholders can directly supervise and control the behavior of the management to ensure that the strategic behaviors such as investment decision-making take the sustainable development as the core and run in the direction that meets the expectations of major shareholders. Enterprises with higher centrality occupy a more important position in the entire interlocking directorate network, and their exposure will increase with the enhancement of centrality. Affected by the “reputation mechanism,” enterprises, as “public stars,” tend to actively respond to the pressure of social CSR, and attach importance to the suggestions provided by interlocking directorates. With the advantage of direct-control right, major shareholders promote the fulfillment of CSR by participating in the decision-making or supervising the management, so as to maintain a good relationship with stakeholders, form a good corporate reputation, improve the ability to obtain resources and finally promote the long-term development of the enterprise. On the one hand, the social reputation recognized by the peers will accumulate a wider network of contacts, open up more information channels, and obtain better career prospects and more board seats for the major shareholders (Engelen and Neumann, 2016). On the other hand, in terms of corporate image, major shareholders are the “image spokesperson” of the company, and the image of shareholders also represents the image of the company. Now, the personal reputation of major shareholders is tied to the reputation of the organization, forming the “reputation duplicate effect,” which further urges enterprises to fulfill their social CSR.

However, from the perspective of “entrenchment effect,” the major shareholders will be driven by their own short-term opportunistic behavior, and regard CSR as a series of behaviors that cannot obtain returns in short term, but constitutes corporate expenditure when the equity is highly concentrated (Waddock, 1997). Therefore, they are not willing to pay too much attention or even ignore this kind of behaviors. Because of the absolute controlling rights, major shareholders overhead the rights and interests of minor shareholders (Claessens and Djankov, 2000); thus, led the enterprises lose the diversity of decision-making of the board of directors, aggravate the agency problem and reduce the decision-making efficiency of the board of directors. When an enterprise is in the center of social network, it means that the operation of this enterprise is connected with many related partners. As the centrality of interlocking directorate network can enhance the coordination and cooperation among organizations, the probability of damage to enterprises caused by the capital flow breakdown and the impact of environmental change of high centrality enterprises is greater compared with enterprises with low centrality. That is, the economic interests of major shareholders are damaged, which is obviously not in line with the expectations of major shareholders. To ensure that enterprises can cope with the impact of the capital flow breakdown and environmental change, major shareholders will instead invest in projects that increase the economic benefits of enterprises and reduce the “useless” ability of CSR.

**Hypothesis 3a:** Ownership concentration moderates the positive relationship between the centrality of interlocking directorate network and CSR, such that the position relationship is stronger when ownership concentration is higher.

**Hypothesis 3b:** Ownership concentration moderates the negative relationship between the centrality of interlocking directorate network and CSR, such that the position relationship is weaker when ownership concentration is higher.

CSR is a signal that enterprises are constantly transmitting good economic operation and development prospects to stakeholders in the external economic environment. The establishment of this good image silently attracts customers, potential investors, employees, the public and the media, and constructs the competitive advantage of the enterprise to a certain extent (Brammer, 2008). Based on the incentive effect of large shareholders, when the ownership concentration is high, the interests of major shareholders are closely related to the prosperity and loss of the enterprise. Therefore, major shareholders often have “interest linkage effect” with enterprises in this situation, so as to urge enterprises to improve legitimacy through CSR. In addition, affected by the “embeddedness mechanism” of social network, whether enterprises can maintain good competitiveness in the market depends on the resources of their stakeholders (Pfeffer and Salancik, 1976), which makes enterprises with rich structural holes actively fulfill CSR to obtain the resources and trust of peripheral companies.

However, no matter how abundant the resources in the social network relationship are, it will still cause adverse effects if there is no reasonable supervision and utilization (Granovetter, 1985). Being in the position of the structural holes means that the enterprises have the control ability and intermediary ability (Burt, 2000). Compared with high network centrality enterprises with dense networks and redundant information, the networks around companies in the structural hole position are sparse, but this kind of enterprises have strong right of information control, and its dependence on the resources of its affiliated enterprises and the government will become weaker (Reitz, 1979). These stakeholders are the source of the pressure of CSR. In other words, the monopoly of their own resources will reduce the pressure on the legitimacy of enterprises in the social environment (Battilana, 2012). The information advantage of structural hole enables the enterprises who are in this position to have preferential access to implicit and unique resources. Driven by selfish nature and interests, major shareholders often choose to monopolize resources to avoid supervision, and only support behaviors and decisions that are obviously beneficial to the economic benefits of the company.

**Hypothesis 4a:** Ownership concentration moderates the positive relationship between the structural hole of interlocking directorate network and CSR, such that the position relationship is stronger when ownership concentration is higher.

**Hypothesis 4b:** Ownership concentration moderates the negative relationship between the structural hole of interlocking directorate network and CSR, such that the position relationship is weaker when ownership concentration is higher.

## Materials and Methods

### Source of Data

We mainly take the China's A-share listed companies from 2010 to 2019 as the research samples, and take data from China Stock Market & Accounting Research Database (CSMAR) database and corporate responsibility rating sores of Hexun.com as the main data sources, which covers a total of 4,042 listed companies. The original data of the interlocking directorate network comes from the basic information of the company executives in the part of governance structure of CSMAR database. Then, Pajek software is used to process the original data to obtain the characteristic index of the interlocking directorate network. To ensure the validity of the study, the initial samples are deleted according to the following conditions. First, excluding sample enterprises with ST at any time during 2011–2019; second, excluding sample enterprises that were delisted at any time from 2011 to 2019; third, excluding sample enterprises with missing core variables. Finally, a total of 9,931 effective observations of 1,034 companies in 10 years are obtained, accounting for 25.58% of the original sample enterprises.

### Measures

#### Corporate Social Responsibility

At present, the measurement methods of CSR mainly include content analysis method, reputation index method, professional agency rating method, and KLD index, which is an evaluation index of CSR proposed by Kinder, Lydenberg and Domini (Zhou et al., 2016; Park and Jeun, 2019; Joo, 2020). The professional agency rating method is used commonly, which is mainly measured by corporate responsibility score of “Hexun.com” or Runling global corporate responsibility rating. The data about CSR in this study uses the method of Python to capture the score of CSR from the social responsibility scoring standard of listed companies published by Hexun.com. The professional evaluation system of CSR report of listed companies of Hexun.com is divided into the following five aspects: Shareholder responsibility, employee responsibility, supplier, customer and consumer responsibility, environmental responsibility, and social responsibility. We use the total score of the five aspects to represent the corporate's degree of CSR.

The dimension division of CSR in this study is based on the view of triple bottom line (Park and Jeun, 2019). According to this basis, shareholder responsibility, employee responsibility, supplier, customer and consumer responsibility belong to the responsibility of direct interests, which is defined as economic CSR in this study. The data is from the sum of the rating scores of the three kinds of responsibility in Hexun.com.

Different from economic CSR, “Hexun.com” distinguishes industries when scoring environmental CSR, and focuses on different scoring standards for manufacturing and service industries in environmental and social CSR. Specifically, for the manufacturing industry, the environmental CSR is empowered by 30% and the social CSR is 10%; for the service industry, the environmental CSR is empowered by 10% and the social CSR is 30%; for other industries, the environmental CSR and social CSR are both empowered by 20%. Considering the accuracy of the data, we calculate the weight according to the industry of the enterprises, and get the environmental CSR and social CSR scores of each enterprise.

#### Centrality

Network centrality includes degree centrality, closeness centrality and betweenness centrality (Freeman, 1978). Degree centrality describes the number of individuals who have direct connections in the network, and reflects the ability of the actor to interact with other actors (Hochbergy et al., 2007); closeness centrality takes the “distance” between the enterprise and other member enterprises in the network as the measurement index to measure the speed of information flow in the network; and betweenness centrality takes the degree that an enterprise in the network is between any other two member enterprises as the measurement standard to investigate the intermediary position of enterprises in the network (Burt, 1992). Specifically, degree centrality indicates the connection between the enterprise and other enterprises in the network. The higher degree centrality means the more individuals directly associated with the enterprise, which depicts the activity and visibility of the enterprise in interlocking directorate network (Fan et al., 2021). Therefore, given the practice of Dijkstra (1959) and Hochbergy et al. (2007), we use degree centrality to measure the centrality of interlocking directorate network. The specific calculation formula is as follows:


$$\begin{array}{l} Degree_{m}=\frac{\sum_{}^{}m\neq nX_{mn}}{g-1} \end{array}$$

where *m* refers to one of the directors of the enterprise; *n* refers to one of the other directors except for *m* director; *g* refers to the total number of board of directors in that year; *X*_*mn*_ refers to a network tie, which is 1 when at least one tie exists between director *m* and director *n*; otherwise, it is 0.

Before calculating the degree centrality of interlocking directorate network, we firstly collect and sort out the data of the enterprises' shareholders who are also shareholders of other listed enterprises from CSMAR database, and form the 2-mode data of “company-director.” Then, we convert the 2-mode data into a 1-mode matrix of “company × company” by the social network analysis software “Pajek.” Finally, we calculate the degree centrality and use the maximum degree of centrality to represent the social network centrality.

#### Structural Hole

The content of structural holes includes effective size, efficiency, constraint, and hierarchy. The constraint is the most important, which reflects the ability of individuals to use structural holes in the network. It also is the mainstream measurement of calculating structural (Burt, 1992). Constraint can effectively measure the lack of structural holes, and the higher the degree of constraint, the fewer structural holes the enterprise has. Based on the practice of Zaheer (2005), we use “1—constraint” to measure the richness of structural holes. The specific calculation formula is as follows:


$$\begin{array}{l} {SH}_{AB}={\text{1}-\underset{B}{\sum }\left(P_{AB}+\underset{C}{\sum }P_{AC}P_{CB}\right)}^{\text{2}}\left(C\neq A,B\right) \end{array}$$

where *P*_*AB*_ indicates the strength of direct relationship between company A and company B; *P*_*AC*_ and *P*_*CB*_ indicate the strength of indirect relationship between company A and company B, respectively, through company C; $P_{AB}+\underset{C}{\sum }P_{AC}P_{CB}$ indicates the sum of all the direct and the indirect relationships between company A and company B. The larger the difference of the formula, the richer the structural holes in interlocking directorate network.

#### Ownership Concentration

Generally, the measurement of ownership concentration mostly selects the sum of the shareholding proportion of the first major shareholder, the shareholding proportion of the top three shareholders and the shareholding proportion of the top 10 shareholders as the evaluation index (Li et al., 2015b; Wang et al., 2019a; Rojahn, 2022), which show the distribution and concentration of the company's equity. The smaller the numerical value of the index is, the more dispersed the equity is. On the contrary, the larger the numerical value is, the more concentrated the equity is. Referring to the common practice, we take the sum of the shareholding proportion of the top three shareholders as the measurement index of ownership concentration.

#### Control Variables

Based on the practices of Desender et al. (2013) and Peng (2014), we control the variables that may affect CSR in corporate characteristics and corporate governance: company size, risk, financial performance, nature of equity, profitability and liquidity ratio at the level of company characteristics, and board size, number of independent directors and CEO duality at the level of corporate governance. In addition, we also control the possible impact of the industry and year.

All variables and their descriptions are summarized below. Table 1 lists all the variables.

**Table 1:** Meaning of variables.

**Type**	**Name**	**Symbol**	**Formula**
Dependent	CSR	CSR	Comprehensive score of CSR from Hexun.com
	Economic CSR	EC	Score of economic CSR/weight from Hexun.com
	Environmental CSR	EN	score of environmental CSR/weight from Hexun.com
	Social CSR	SC	score of social CSR/weight from Hexun.com
Independent	centrality structural hole	DC SH	Degree centrality “1−constraint”
Moderating variables	Ownership concentration	OC	The sum of the shareholding proportion of the top three shareholders
Controls	Size	Size	ln (total assets at the end of the year)
	Risk	Risk	ln (total liabilities/total assets)
	Financial performance	roe	Net profit/net assets ×100%
	Nature of equity	st	State-owned enterprise is 1; otherwise, it is 0
	Profitability	*P*	Net profit/total income
	Liquidity ratio	L	Total current assets/total assets
	Board size	Board	Total number of directors
	Independent directors	ID	The number of independent directors
	CEO duality	DU	Chairman and CEO is one of the two staff, is 0; otherwise, it is 1
	Industry	Ind	17 dummy variables
	Year	Year	10 dummy variables

*Source from the author's collection*.

### Methods

To test the hypotheses discussed here, we use STATA to conduct multiple regression to explore the impact of interlocking directorate network position on CSR, and adds year and industry fixed effect to control the characteristics of changes over time and different industries. Given that all variables were collected at the firm level, data quality was analyzed prior to regression to ensure that the data were normally distributed. In the test of moderating effect, the interaction is introduced and the individual effect is controlled to identify the significance of moderating effect.

## Results

### Descriptive Statistics and Correlation Analysis

Table 2 presents means, standard deviations, and correlations. The magnitude of the correlations indicates that multicollinearity is not a serious problem. The results are generally consistent with the hypotheses discussed here: The network position of corporate plays a positive role in promoting CSR, and has a stronger role in promoting economic CSR and environmental CSR. Specifically, the centrality and structural hole of interlocking directorate network are significantly positively correlated with the variables of CSR and its three dimensions (economic CSR, environmental CSR, and social CSR) at the confidence level of 1%, and the correlation coefficients are 0.23, 0.23, 0.08, 0.34, 0.28, and 0.16, respectively. Therefore, there is a significant correlation between the variables studied in this study. In addition, the magnitude of Variance Inflation Factor (VIF) is less than 10^1^ (Kalnins, 2018), which indicates that multicollinearity is not a serious problem.

**Table 2:** Results of descriptive statistics and multicollinearity test.

	**Mean**	**Std**.	**1**	**2**	**3**	**4**	**5**	**6**	**7**	**8**	**9**	**10**	**11**	**12**	**13**	**14**	**15**	**16**
1.CSR	24.68	16.46	1.00															
2.EC	30.21	17.93	0.96	1.00														
3.EN	7.34	20.19	0.82	0.74	1.00													
4.SC	36.55	32.17	0.39	0.23	0.04	1.00												
5.DC	10.54	7.40	0.25	0.23	0.23	0.08	1.00											
6.SH	0.62	0.24	0.35	0.34	0.28	0.16	0.10	1.00										
7.OC	−0.91	0.60	0.95	0.91	0.77	0.37	0.23	0.34	1.00									
8.risk	22.49	1.42	0.01	−0.07	0.10	−0.02	0.03	0.00	0.00	1.00								
9.size	0.04	0.52	0.29	0.28	0.20	0.08	0.08	0.10	0.27	0.45	1.00							
10.roe	8.83	1.78	0.15	0.18	0.02	0.10	0.02	0.06	0.14	−0.07	0.04	1.00						
11.board	0.41	0.17	0.13	0.13	0.13	0.02	0.02	0.06	0.12	0.12	0.25	0.02	1.00					
12.st	0.09	0.82	0.14	0.10	0.15	0.03	0.03	0.06	0.13	0.27	0.32	0.00	0.25	1.00				
13.P	0.55	0.21	0.10	0.13	0.02	0.04	0.01	0.04	0.11	−0.04	0.09	0.12	0.01	0.01	1.00			
14.L	3.88	1.21	0.03	0.03	−0.08	0.12	−0.01	0.01	0.03	−0.07	−0.15	0.05	−0.15	−0.14	0.00	1.00		
15.ID	0.77	0.42	0.03	0.04	0.03	0.00	−0.01	0.02	0.03	0.10	0.20	−0.01	0.41	0.13	0.00	−0.10	1.00	
16.DU	0.47	0.50	0.06	0.04	0.07	0.01	0.02	0.02	0.06	0.13	0.14	−0.02	0.16	0.29	0.01	−0.09	0.06	1.00

### Hypothesis Testing

#### Test of Main Effects

Table 3 presents the basic regression results on how network position affects CSR. Specifically, the regression coefficients of the centrality and structural hole of interlocking directorate network for CSR are 0.415 and 18.675 respectively, which indicates that there is a significant positive impact at the confidence level of 1%, supporting Hypothesis 1a and Hypothesis 1b. Columns marked as Model 2A, Model 2B, Model 3A, Model 3B, Model 4A, and Model 4B in Table 3 show the effects of the centrality and structural hole of interlocking directorate network on economic CSR, environmental CSR, and social CSR. The results show that, consistent with Hypothesis 2, the network position of corporate has a different impact on CSR in the three dimensions.

**Table 3:** Results of regression analysis for interlocking directorate network position and CSR.

**Var**	**CSR**		**EC**		**EN**		**SC**	
	**Model 1A**	**Model 1B**	**Model 2A**	**Model 2B**	**Model 3A**	**Model 3B**	**Model 4A**	**Model 4A**
DC	0.415*** (19.37)		0.412*** (17.84)		0.495*** (16.58)		0.298*** (7.54)	
SH		18.675*** (34.14)		19.162*** (32.21)		18.761*** (27.78)		17.417*** (12.14)
Risk	−4.669*** (−17.11)	−4.276*** (−16.34)	−7.11*** (−22.78)	−6.706*** (−22.35)	−0.221 (−0.70)	0.160 (0.51)	−3.930*** (−6.25)	−3.547*** (−5.72)
Size	5.053*** (33.85)	4.843*** (33.87)	5.961*** (37.30)	5.736*** (37.23)	3.944*** (19.29)	3.784*** (19.06)	3.541*** (11.90)	3.286*** (11.13)
Board	0.325** (3.22)	0.261** (2.69)	−0.392*** (3.57)	0.327** (3.10)	0.272* (2.10)	0.206 (1.62)	0.052 (0.24)	−0.005 (-0.02)
Roe	2.558** (3.01)	2.311** (2.97)	3.495** (3.21)	3.240** (3.18)	−0.464 (−1.28)	−0.702* (−1.99)	4.767*** (3.52)	4.524*** (3.58)
St	1.032** (3.01)	0.779* (2.47)	0.407 (1.17)	0.150 (0.44)	1.843*** (4.46)	1.578*** (3.86)	1.881** (2.49)	1.659* (2.21)
P	1.343** (3.05)	1.209** (3.09)	1.868** (3.15)	1.730** (3.19)	0.029 (0.09)	−0.104 (−0.31)	1.607* (2.40)	1.480* (2.36)
L	4.929*** (5.73)	4.586*** (5.49)	6.750*** (7.22)	6.399*** (7.04)	−2.948** (−2.67)	−3.292** (−3.02)	13.577*** (7.39)	13.258*** (7.26)
ID	−0.373** (−2.74)	−0.406** (−3.08)	−0.403** (−2.70)	−0.436** (−3.01)	−0.350* (−2.02)	−0.394* (−2.31)	−0.193 (−0.63)	−0.212 (−0.07)
DU	0.535 (1.61)	0.673* (2.08)	0.301 (0.82)	0.440 (1.23)	0.593 (1.49)	0.748 (1.90)	1.961* (2.47)	2.071* (2.62)
Ind	YES	YES	YES	YES	YES	YES	YES	YES
Year	YES	YES	YES	YES	YES	YES	YES	YES
Obs	9,684	9,684	9,684	9,684	9,684	9,684	9,684	9,684
*R* ^2^	0.2953	0.3334	0.3015	0.3374	0.2193	0.2351	0.1020	0.1138

t-values are in parentheses; ***p < 0.001, **p < 0.01, *p < 0.05.

It can also be seen from Table 3 that network centrality and structural holes have a more significant impact on economic CSR and environmental CSR, which may be due to the fact that economic CSR and environmental CSR belong to basic responsibility and they are related to the direct stakeholders of the enterprises and the interests that directly affect firm performance. All enterprises pursue profit maximization, and the economic CSR is easier to meet the economic benefits of enterprises and the fulfillment of environmental CSR will make it easier for enterprises to obtain the favor of stakeholders. By contrast, social CSR is a kind of high-level responsibility, which is more based on empathy and moral constraints. It is closer to the moral level, which is high-level responsibilities without mandatory provisions. Enterprises can be praised for their active performance, but will not be punished by laws and regulations and criticized by the public opinion if they do not actively fulfill this kind of responsibilities. Therefore, compared with social CSR, enterprises often take more positive response measures to economic CSR and environmental CSR.

In addition, we also carried out standardized regression on the original models and obtained the standardized regression coefficients. The results also indicate that there is a positive effect of the centrality and structural hole of interlocking directorate network on the level of CSR, and the positive effects on economic CSR and environmental CSR are stronger compared with social CSR. The details are shown in Appendix.

Considering the robustness of the results discussed here and ensure the reliability and authenticity of the research conclusions, the robustness test is carried out by changing the regression model. In the robustness test, we perform regression on the characteristic index of interlocking directorate network with a lag of one period, and test the impact of network centrality and structural holes on CSR. The results in Table 4 show that the research conclusions will not be disturbed by the reverse causality.

**Table 4:** Results of robustness.

**Var**	**CSR**		**EC**		**EN**		**SC**	
lag_DC	0.219*** (9.74)		0.229*** (9.47)		0.267*** (9.04)		0.065* (2.52)	
lag_SH		9.770*** (16.01)		10.740*** (16.18)		9.844*** (13.25)		4.697** (3.22)
Control	YES	YES	YES	YES	YES	YES	YES	YES
Ind	YES	YES	YES	YES	YES	YES	YES	YES
Year	YES	YES	YES	YES	YES	YES	YES	YES
obs	8,504	8,504	8,504	8,504	8,504	8,504	8,504	8,504
*R* ^2^	0.2619	0.2724	0.2780	0.2896	0.1767	0.1808	0.0960	0.0970

t-values are in parentheses; ***p < 0.001, **p < 0.01, *p < 0.05.

#### Test of Moderating Effects

Consistent with Hypothesis 3a, the interaction coefficient between network centrality and ownership concentration (OC_DC = 0.121, *p* < 0.05) is significantly positive in Table 5, indicating that the ownership concentration of enterprises positively moderates the relationship between network centrality and the level of CSR. The higher the level of ownership concentration, the stronger the positive relationship between the network centrality of the enterprise and the level of CSR. At this time, the incentive effect of shareholders is dominant, and the “reputation superposition effect” is confirmed. In addition, the interaction coefficient between structural holes and ownership concentration (OC_SH = 16.310, *p* < 0.001) is also significantly positive, which shows that the ownership concentration of enterprises positively moderates the relationship between network structural holes and the level of CSR. Hypothesis 4a is supported. Therefore, it is concluded that the higher the level of ownership concentration, the stronger the positive relationship between the structural holes and CSR, and the “interest linkage effect” has been effectively confirmed.

**Table 5:** Results of moderating effect.

		**CSR**		
DC	0.415*** (19.37)	0.013 (0.66)		
SH			18.675*** (34.14)	−3.233*** (−5.36)
OC		88.137*** (112.35)		77.026*** (60.97)
OC_DC		0.121* (2.37)		
OC_SH				16.310*** (9.37)
Control variable	YES	YES	YES	YES
Ind	YES	YES	YES	YES
Year	YES	YES	YES	YES
*R* ^2^	0.2953	0.9047	0.3334	0.9061

t-values are in parentheses; ***p < 0.001, **p < 0.01, *p < 0.05.

### Further Study

The whole samples are classified according to the nature of property rights and industries to test the impact of the characteristics of interlocking directorate network on the level of CSR respectively. The results show that the network centrality and the structural holes have more significant impacts on the CSR of state-owned enterprises than that of private enterprises. This may because the ownership of state-owned enterprises is owned by the state, which has a stronger exemplary effect and plays an exemplary role than private enterprises. In addition, the positive impact of characteristics of interlocking directorate network on the level of CSR in manufacturing industry is significantly stronger than that in Information Technology (IT) enterprises. This is because the products of IT enterprises are more dependent on their technical and scientific content, rather than relying too much on reputation mechanism and resources of stakeholders. In contrast, manufacturing enterprises have strong substitutability, so they rely more on corporate image to gain the preferences of the public, and their resource dependence on stakeholders is far stronger than that of IT enterprises. Therefore, the characteristics of interlocking directorate network in manufacturing enterprises have a more significant positive impact on the fulfillment of CSR.

## Discussion

Based on social network theory and institutional theory, this study examines the impact of director network position on CSR. Due to the heterogeneity of information and resources transmitted by network centrality and structural holes, the research focuses on the impact of the two aspects on CSR. In addition, it also analyzes the different performances of interlocking directorates in economic CSR, social CSR, and environmental CSR. Finally, the moderating effect of ownership concentration on this effect is tested. By analyzing the data of 1,034 Chinese listed companies for 10 years, the results show that the higher the centrality and structural hole of interlocking directorate network, the more actively the enterprises can fulfill their social CSR, and have a deeper impact on environmental CSR. In addition, the ownership concentration positively moderates the relationship between the network position and CSR. The stronger the ownership concentration, the greater the impact of the network position on CSR. Through the investigation, the results have theoretical and practical significance for the future research. The results of this study provide a richer perspective for social network theory and institutional theory. In the context of social network, the legitimacy recognition of institutional theory is one of the necessary conditions for the survival and development of enterprises. Enterprises will obtain the continuous inflow of key information and resources in the network by undertaking CSR, so as to obtain the legitimacy recognition. They obtain the continuous resources and then reduce the impact of the environment and promote investment and cooperation. This will have theoretical and practical significance for the future research.

### Theoretical Contributions

This study has made the following contributions to the related research of CSR. First, the research reveals the relationship between the network position and CSR from the aspects of centrality and structural holes, which enriches the research on the effectiveness of CSR at the level of social network. Although more and more studies emphasize the importance of interlocking directorate network (Cai et al., 2014; Howard and Withers, 2017), few studies pay attention to the relationship between interlocking directorate network and different dimensions of CSR. Through empirical analysis, this study preliminarily investigates the impact of the characteristics of interlocking directorate network on CSR, and also enriches the research corporate governance in the field of social norms.

Second, this study emphasizes the impact of network position on different dimensions of CSR, and the empirical test shows that the characteristics of interlocking directorate network have a more significant impact on economic CSR and environmental CSR compared with social CSR. This situation shows that under the background of China's economic transformation, enterprises form a community of interests through the interlocking directorate network so as to reduce transaction costs and improve communication efficiency (Shipilov and Greve, 2010). For the environmental CSR and economic CSR, which are helpful to increase the possibility of cooperation with other enterprises and are in an increasingly important position, the enterprises will put their energy into the fulfillment of these kinds of CSR, while for the high-level social CSR such as charitable donation, which may have adverse effects on the economic interests of the enterprises, the enterprises will not show a particularly positive attitude.

Third, from the perspective of ownership structure, the study also reveals the boundary conditions of the impact of network position on CSR. We put forward the positive and negative hypothesis based on the incentive effect and entrenchment effect of major shareholders when studying the moderating effect of ownership concentration. Previous studies rarely consider the effects of the two effects at the same time, but this study explains the mechanism of the moderating effect through the two effects, which enriches the existing research. Specifically, the results show that the higher the ownership concentration of enterprises, the stronger the positive impact of network position on CSR. The research of enterprise ownership concentration complements the contingency of the impact of social network position on CSR. From this perspective, it can be inferred that the future research can start with other boundary conditions of social network position to explore the impact on CSR.

### Practical Implications

The study has important implications for enterprise practice as well. At first, as the external governance mechanism of the company, the centrality and structural hole of interlocking directorate network can be designed and changed for enterprises. We can give full play to the governance mechanism of the interlocking directorate network. Existing researches show that the characteristics of directorate network will have a positive impact on CSR. Therefore, enterprises should make full use of it based on the advantages of social capital brought by the informal system (Cheung et al., 2013). When an enterprise is in the period of transition, interlocking directorates are more able to observe the impact of external environmental changes on the enterprise, and fully participating in the network can effectively alleviate the external impact (Parsons and Sulaeman, 2018). At the same time, the appointment of interlocking directorates is also one of the important tasks: interlocking directorates with “high-quality” and “good reputation” will bring enterprises huge value. On the contrary, board of directors with “low-quality” virtually breaks the whole interlocking directorate network of the company, causing huge losses. Absolutely, enterprises should employ interlocking directorates and layout their position reasonably in the social network. CSR should be a polishing tool of enterprises to perform within their own capabilities.

Next, the research results highlight the impact of corporate ownership concentration on the relationship between network position and CSR, which indicates that the rational allocation of shareholder power and management power is also noteworthy (Buertey, 2021). When shareholders hold the decision-making power alone, it will affect the decision-making of the board of directors and the response measures of executives to market changes, resulting in short-term opportunistic behavior (Anderson, 2003); If the executive power is too large, it will overhead the power of shareholders, resulting in damage to shareholders' rights and interests (Adams and Licht, 2011), which will also affect the value of the enterprise. When shareholders have the right to make decisions alone, it will affect the decisions of the board of directors and the executives' response measures to market changes, resulting in short-term opportunistic behavior. However, if the executives' power is too great, it will overhead the power of shareholders and damage shareholders' rights and interests, which will also affect the enterprise value. Briefly, shareholders and executives can carry out effective supervision and play a positive role in promoting enterprise development only when the two kinds of powers are fully checked and balanced.

### Limitations and Future Directions

Generally speaking, the research supports the argument that the characteristics of interlocking directorate network have a positive impact on the level of CSR in theoretical and empirically, and has been verified in three dimensions of CSR, but there are still some defects in the research.

First, there are many factors that affect the level of CSR. It is not enough to explore the factors only from the level of external governance mechanism. The joint efforts of the government and enterprises are needed to form a good CSR atmosphere. It is better to formulate a series of policy measures to guide enterprises to fulfill their social responsibilities and promote outstanding demonstration enterprises to share successful experiences (Lin, 2010). Second, we only consider the interlocking directorate network among enterprises, but there are still many ways for enterprises to form social networks. Therefore, whether the conclusions of this study are established in other social networks remains to be further studied, and it should be analyzed in combination with various network forms in the future. Third, the research adopts the CSR score from “Hexun.com,” which depends on whether the social responsibility report disclosed by the enterprise is accurate. Although the rating is relatively authoritative in Hexun.com, the accuracy, omission and lack of data will still have a certain impact on the research results.

The fulfillment of CSR is a long-term development process, which requires the joint efforts of enterprises and society. The practice of CSR in China is in an exploratory period, and there is no systematic conclusion yet. Therefore, the research on CSR needs to be expanded. In addition, the rational allocation of shareholder power and management power is also noteworthy. The concept of separation of powers and checks and balances has always occupied the mainstream position. The power of enterprise shareholders, board of directors and management should be reasonably allocated. The imbalance of rights will inevitably damage the interests of enterprises. Both sides carry out effective supervision only when the powers are fully checked and balanced. Therefore, it is better to focus on the impact of the company's central position in the ownership network on CSR, and explore the direct impact of corporate ownership structure on social responsibility behavior.

## Data Availability Statement

Publicly available datasets were analyzed in this study. This data can be found at: https://www.gtarsc.com; https://www.hexun.com.

## Author Contributions

LQ, YX, and YG completed the research design together. LQ provided research assistance and support. YX and YG collected and analyzed the data and wrote the manuscript. All authors contributed to manuscript revision and agreed to publish the study.

## Funding

This study was supported by the National Social Science Foundation of China (Grant No. 19AGL015).

## Conflict of Interest

The authors declare that the research was conducted in the absence of any commercial or financial relationships that could be construed as a potential conflict of interest.

## Publisher's Note

All claims expressed in this article are solely those of the authors and do not necessarily represent those of their affiliated organizations, or those of the publisher, the editors and the reviewers. Any product that may be evaluated in this article, or claim that may be made by its manufacturer, is not guaranteed or endorsed by the publisher.

## Footnotes

^1^When VIF < 10, it indicates that there is no multicollinearity.

## Appendix

**TABLE A1:** Results of standardized regression analysis for corporate network position and CSR.

**Var**	**CSR**		**EC**		**EN**		**SC**	
DC	0.187*** (19.37)		0.171*** (17.84)		0.181*** (16.58)		0.069*** (7.54)	
SH		0.274*** (34.14)		0.258*** (32.21)		0.222*** (27.78)		0.130*** (12.14)
Controls	YES	YES	YES	YES	YES	YES	YES	YES
Ind	YES	YES	YES	YES	YES	YES	YES	YES
Year	YES	YES	YES	YES	YES	YES	YES	YES
obs	9,684	9,684	9,684	9,684	9,684	9,684	9,684	9,684
	0.2953	0.3334	0.3015	0.3374	0.2193	0.2351	0.102	0.114

*t-values are in parentheses; ***p < 0.001, **p < 0.01, *p < 0.05*.
